# Turning losses into gain in nonlinear optics

**DOI:** 10.1038/s41377-018-0077-y

**Published:** 2018-10-17

**Authors:** Franco Prati

**Affiliations:** Department of Science and High Technology University of Insubria, Como, 22100 Italy

Modulational instabilities in nonlinear optics are the fundamental processes that cause the transition from uniform states to structures in extended systems. Quite counterintuitively, such processes can be enhanced rather than inhibited when spectrally asymmetric losses are introduced into the system.

In his fragment 76, the philosopher Heraclitus wrote “The death of earth is to become water, and the death of water is to become air, and the death of air is to become fire, and reversely”^1^. This sentence describes well the “identity of the opposites”, one of the most fruitful principles of dialectic thinking from ancient Greece to Hegel and beyond. These thoughts came to my mind when I read a recent paper by Perego and coworkers^2^, in which they show how in nonlinear optical systems, spectrally asymmetric losses can generate gain in the very same beam that is affected by losses. The death of losses is to become gain.

Perego et al. name this phenomenon “gain through losses” (GTL). It affects several types of modulational instabilities^3^, making them possible even in regimes in which the system would be stable in absence of asymmetric losses. Modulational instabilities in physics, and specifically in nonlinear optics, are known to give birth to the most diverse spatio-temporal dynamics, ranging from regular trains of pulses to turbulence. The most relevant instabilities are associated with the names of Benjamin and Feir, Faraday, and Turing and paradigmatic amplitude equations such as the nonlinear Schroedinger, the complex Ginzburg-Landau and the reaction-diffusion equations, which are reviewed in a comprehensive manner by Perego et al. in the first part of the paper.

When an optical system is modulationally unstable, it experiences growth of modes (sidemodes) whose frequencies are symmetric with respect to that of the driving field, which feeds the system with energy. Then, the energy possibly propagates to other adjacent modes through four-wave mixing processes, giving rise to complex nonlinear structures. In this scheme, it is obvious that the introduction of (symmetric) linear losses into the unstable sidemodes has the effect of reducing their linear growth rate, if not totally inhibiting the instability.

**Fig. 1:**
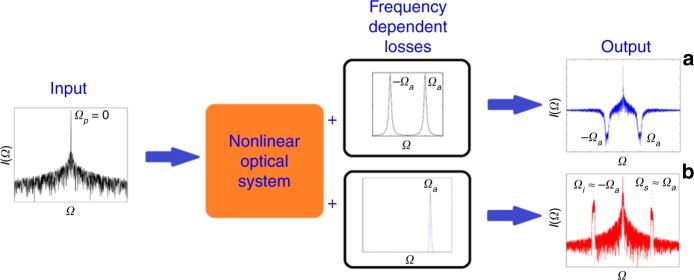
Schematic view of the “gain through losses” principle. A nonlinear system is driven by a coherent pump field at the reference frequency *Ω*_*p*_ = 0 and suffers from frequency-dependent losses. In **a**, the losses are centered at the symmetric frequencies −*Ω*_a_ and *Ω*_a_, and if sufficiently large, they inhibit the growth of the sidemodes at those frequencies even if the system is modulationally unstable. In **b**, the losses are centered around the frequency of one of the two sidemodes, and this causes the growth of both sidemodes even if the system is modulationally stable in the absence of losses

What Perego et al. show is that under certain conditions, the introduction of *asymmetric losses* into two sidemodes can favor the growth of such modes by subtracting energy from the driving field (Fig. 1). This quite counterintuitive idea (the power of dialectic thinking) is validated by three specific examples.

*GTL-based fiber amplifier*: it has been known since the 1960s that optical fibers support bright solitons as a balance of *anomalous* group velocity dispersion (GVD) and self-phase modulation due to the Kerr effect^4^. Perego et al. consider instead the regime of *normal* GVD and the effects of a generic two-level atoms absorber acting as a spectrally asymmetric filter satisfying the Kramers-Kronig relations. The system is described by a generalized nonlinear Schroedinger equation, and a simple stability analysis reveals that the sidemodes resonant with the central frequencies of the absorber are amplified through a process that is more efficient when the sidemodes are closer to the driving field.

*Pulses and frequency combs in a ring resonator*: a similar system, which has been attracting a large amount of attention in recent years because of its connection with frequency combs, is the driven ring fiber resonator described by the Lugiato-Lefever equation (LLE)^5^. In the anomalous dispersion regime, the LLE presents a Turing instability, which gives rise to trains of pulses or isolated structures, called cavity solitons, which are the counterpart of frequency combs in the time domain. Again, Perego et al. show that if a lumped asymmetric spectral filter is inserted into the cavity, trains of pulses can be generated even in the normal dispersion regime, in which the standard LLE predicts only the existence of dark solitons.

*GTL in optical parametric oscillators*: in an optical parametric oscillator, amplification of the sidemodes is realized parametrically, i.e., without transferring atoms from a lower to a higher energy level. The maximum efficiency is achieved for perfect phase matching, a condition that is difficult to realize experimentally, and even a small deviation from that condition may cancel the amplification. Perego et al. show that GTL allows enlarging the interval of acceptable phase mismatch, making possible parametric amplification even in cases in which phase matching can be barely achieved.

In the above examples, losses are stationary, but novel modulational instabilities may arise when losses are modulated periodically in time, thus establishing a connection between GTL and the Faraday instability.

Since GTL has been demonstrated in systems that are described by universal equations such as the nonlinear Schroedinger equation and the complex Ginzburg-Landau equation, it would not be surprising if it finds applications in other fields of physics beyond nonlinear optics.
